# Neuroblastomas in Eastern China: a retrospective series study of 275 cases in a regional center

**DOI:** 10.7717/peerj.5665

**Published:** 2018-09-17

**Authors:** Yangyang Ma, Jicui Zheng, Jiayan Feng, Lian Chen, Kuiran Dong, Xianmin Xiao

**Affiliations:** 1 Department of Pathology, Children’s Hospital of Fudan University, Shanghai, China; 2 Department of Surgery, Children’s Hospital of Fudan University, Shanghai, China

**Keywords:** Neuroblastoma, MYCN status, Clinicopathological features, Prognosis, Surgery

## Abstract

**Purpose:**

Most studies on neuroblastoma (NB) have been conducted in Western countries or Japan. The objective of our study was to analyze clinical and pathological features, MYCN status, surgical methods, and prognosis in Chinese NB patients.

**Methods:**

A retrospective, single-center case series study of 275 NBs was implemented. Clinical manifestations, pathological features, MYCN status, and surgical treatment were analyzed. Log-rank test and Cox hazards models were used to assess overall survivals (OSs).

**Results:**

The cohort consisted of 105 females and 170 males, with an age range of five days to 15 years. MYCN amplification was detected in 21.5% of all cases. The median OS was 15.0 months for MYCN amplified group. The five-year OS rates were 70.8% and 18.3% for MYCN unamplified and amplified groups, respectively, and the comparison of Kaplan–Meier curves for these two groups showed statistical significance (*P* < .001 by log-rank test). Gross total resection (GTR, *n* = 111) and subtotal resection (STR, *n* = 58) were administered in 169 patients at stages 3 and 4 who received chemotherapy and the comparison of Kaplan–Meier curves for different groups in these patients had statistical significance (STR vs. GTR, *P* = .009; MYCN unamplified vs. amplified, *P* < .001 by log-rank test, respectively).The multivariate survival analyses showed statistical significance (STR vs. GTR, *P* = .047; MYCN unamplified vs. amplified, *P* = .001 by Cox regression model).

**Conclusions:**

MYCN amplification is an independently adverse prognostic factor in Chinese NB patients at stages 3 and 4 and GTR is associated with improved OS compared with STR in these patients.

## Introduction

Neuroblastoma (NB) is the most common malignant extracranial solid tumor in children. More than half of pediatric NBs present with disseminated metastases at initial diagnosis, and most of them are in an advanced stage with poor prognoses. Risk assessment in NB depends on the comprehensive analysis of data regarding clinical and biologic characteristics. Children’s Oncology Group (COG) has established the risk stratification system relying on five characteristics: International Neuroblastoma Staging System (INSS) stage, patient age at diagnosis, MYCN gene status, DNA index, and International Neuroblastoma Pathology Classification (INPC) (London et al., 2005; Shimada et al., 1999; Peuchmaur et al., 2003). Specific chromosomal aberrations, such as the 11q loss, have been added to the International Neuroblastoma Risk Group (INRG) classification system (Cohn et al., 2009). According to the COG risk stratification system, three groups were established: low-risk, intermediate-risk, and high-risk groups. Most stage 1 and 2 NB patients (usually in the low-risk group) can be cured by surgery alone and have an excellent prognosis, which has been a consensus worldwide. However, the treatment for stage 3 and 4 NB patients, especially those in the high-risk group, is still tough, and the outcome is dismal. Whether gross total resection (GTR) of the primary tumor can render overall survival (OS) longer in these refractory patients than in those with subtotal tumor resection (STR) is still unclear. Several studies reported that GTR might bring benefits to clinical outcomes in these patients. However, others have drawn opposite conclusions (McGregor et al., 2005; Fischer et al., 2017; Simon et al., 2013; Englum et al., 2015). Studies about clinicopathological features and MYCN status in a cohort of NB patients and the prognosis diversity of different MYCN status and surgical methods (STR vs. GTR) in stage 3 and 4 NB patients in China have rarely been reported.

Herein, we collected 275 NBs between 2010 and 2015 in our center and made a retrospective study of the clinicopathological features, MYCN status, surgical management, follow-up, and prognosis. Our study highlights the prognostic values of MYCN status and different surgical methods in stage 3 and 4 patients in China.

## Materials and Methods

### Medical ethics

This retrospective cohort study was approved by the Ethical Committee of Children’s Hospital of Fudan University (no. 2017-217). The forms of consent were received from the patients’ parents.

### Archive review

Two hundred and seventy-five NBs were retrieved from the medical records of Children’s Hospital of Fudan University between 2010 and 2015. We collected such data as sex, age, chief complaints, lesion site, isotope bone scan, ultrasound imaging, radiological finding, bone marrow biopsy, MYCN status, histopathological diagnosis, INPC, INSS stage, surgical treatment, and follow-up.

MYCN status was evaluated by fluorescence in situ hybridization study and analyzed according to the international consensus by INRG Biology Committee for neuroblastoma molecular diagnostics (Ambros et al., 2009). A >4-fold increase in MYCN signal compared with a reference probe was recognized as MYCN amplification. All the patients were assigned to high-, intermediate-, and low-risk categories according to the COG risk stratification criteria. For those who had finished the treatment course, one outpatient clinic visit for each year was requested. In these visits they were carefully evaluated by physical examination and imaging tests. Patients who failed to visit on time were contacted by phone, mail, and e-mail for collection of data concerning the children’s health and the test results in local hospitals.

### Statistical analysis

All the data were collected and filed in a database. A descriptive analysis was carried out for clinical, pathological, and biological features, surgical management, and follow-up. Statistical analysis was processed by IBM SPSS version 22.0 (IBM Corporation. Armonk, NY, USA). OS was defined as the time from initial diagnosis to death or last follow-up if patients survived. The Kaplan–Meier survival curves were compared with log-rank test. Cox proportional hazards model was used for univariate and multivariate survival analyses. All statistical tests were two-sided and *P* values <0.05 were considered as statistically significant.

## Results

### Clinical characteristics

Clinical and pathological characteristics of 275 cases were summarized in Table 1. The cohort had a slightly male predominance with a male-to-female ratio of 1.6:1. The age range was five days to 15 years. There were 90 infantile NBs. The mean and median ages were 30 months and 23 months, respectively. The two leading primary tumor sites were the adrenal gland and mediastinum (Table 1), and others included retroperitoneal sympathetic chain (*n* = 5), pelvic cavity (*n* = 2), kidney (*n* = 2), neck (*n* = 1), oropharynx (*n* = 1), and orbit (*n* = 1). However, no primary tumor was detected in two cases with metastases in liver and bone marrow, respectively. Six patients had bilateral adrenal neuroblastomas. The most common chief complaints were abdominal mass (88/275), fever (38/275), abdominal pain (37/275), lower extremity pain (26/275), coughing (25/275), and abdominal distention (25/275). Less common symptoms included head and neck mass, skin mass, dyspnea, hepatomegaly, jaundice, and proptosis. The most common distant metastatic sites were bone and bone marrow (Table 1). Other rare metastatic locations included the brain, lung, pleura, orbit, paraspine, pancreas, spleen, and posterior eyeball.

**Table 1:** Clinicopathological features and MYCN status in 275 NBs.

Features	Number	MYCN-amplified	MYCN-unamplified
Sex			
Male	170	39	131
Female	105	20	85
Age(months)			
<18	124	20	104
18–60	102	33	69
≥60	49	6	43
Grade of differentiation			
Undifferentiated	89	37	52
Poorly differentiated	163	22	141
Differentiating	23	0	23
Histology			
Favorable	183	9	174
Unfavorable	92	50	42
INSS stage			
1	44	1	43
2	9	1	8
3	62	11	51
4	143	45	98
4S	17	1	16
Risk group			
Low	55	1	54
Intermediate	54	0	54
High	166	58	108
Primary site			
Adrenal gland	221	54	167
Mediastinum	40	2	38
Others	14	3	11
Distant metastatic site			
Bone	109	28	81
Bone marrow	84	23	61
Lymph node	35	8	27
Liver	36	11	25
Skin	21	6	15
Others	49	19	30

### Pathological characteristics and MYCN status

Pathological diagnosis was determined according to the International Neuroblastoma Pathology Committee system (Peuchmaur et al., 2003; Cohn et al., 2009). The distribution of grade of differentiation and favorable/unfavorable group is listed in Table 1 and poorly differentiated NBs and favorable histology NBs made up the majority of the 275 cases. MYCN amplification accounted for 21.5% of the total cases. Non-MYCN-amplification included MYCN gain (*n* = 7) and wild-type (*n* = 209). MYCN amplification was relatively common for stage 3 and 4 patients, but very rare in stage 1, 2, and 4S patients. The majority of the MYCN-amplified cases appeared in high-risk group (58/59, 98.3%) and UH group (50/59, 84.7%). All the MYCN-amplified cases were either undifferentiated or poorly differentiated subtype. Non-MYCN-amplification was observed in the contemporaneous 77 ganglioneuroblastomas and 55 ganglioneuromas in our center.

### Surgical management, follow-up, and prognosis

The general principles of treatment in our center were surgical resection alone for the low-risk group, surgical resection with chemotherapy for the intermediate-risk group, and multimodal therapy for the high-risk group. This included surgery, chemotherapy, radiation therapy, and autologous stem cell transplantation (ASCT). Anti-GD2 immunotherapy was not used at that time in our center because of its unavailability in China. The chemotherapeutic regimen was consistent with the COG chemotherapy protocols for intermediate-risk group (COG A3961) and high-risk group (COG A3973). Radiation therapy was administered to the high-risk group. ASCT was not widely used in this series because of economic overload and only performed in selectively 14 advanced-stage patients whose parents could afford the costs.

**Figure 1:**
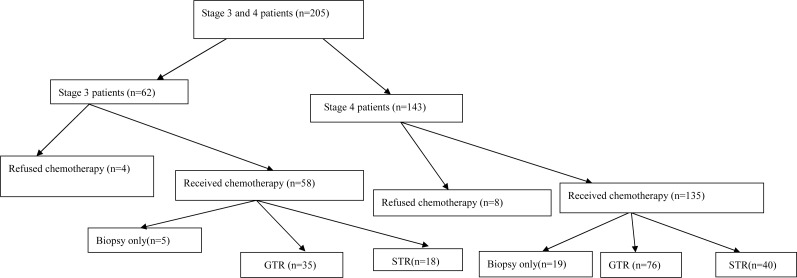
Stage 3 and 4 patient flow diagram.

All the patients at stage 1 and 2 except for two with MYCN amplification (one at each stage) received GTR alone without any other therapy, and were all event-free in the follow-up. In those two patients with MYCN amplification, the tumor recurred a short time after GTR, so chemotherapy was administered and one of the patients also underwent radiation therapy in accordance with high-risk protocols. The GTR rate was 100% for those at stages 1 and 2 in our study. For stage 4S patients, 13 cases including two patients who had the mass enlargement in the wait-and-see approach received GTR of primary tumor and low-dose chemotherapy, three patients decided to wait and see and spontaneous tumor regression was recorded after biopsy, and one patient refused further therapy after biopsy and was lost in the follow-up. The GTR rate was 100% for those at stage 4S who chose to receive surgical removal of primary tumor. Comprehensive therapy was performed for stage 3 and 4 patients who were in the high-risk group. The general situation of stage 3 and 4 patients was frequently worse than that of stage 1 and 2 patients at initial presentation and the adjacent organs and main blood vessels were usually adherent to the tumor or encased by the tumor, which made GTR very difficult. Twenty-four patients at stages 3 and 4 underwent tumor biopsy and chemotherapy without surgical resection. Different surgical methods (GTR or STR) were administered in the rest 169 patients (Fig. 1): GTR (*n* = 111) including primary GTR and delayed GTR after biopsy and chemotherapy; STR (*n* = 58) including primary STR and delayed STR after biopsy and chemotherapy. The GTR rate was 66.0% for stage 3 patients and 65.5% for stage 4 patients whose parents preferred the surgical approach. No surgery-related death was noticed in all the patients. Very few surgical complications were observed.

**Figure 2:**
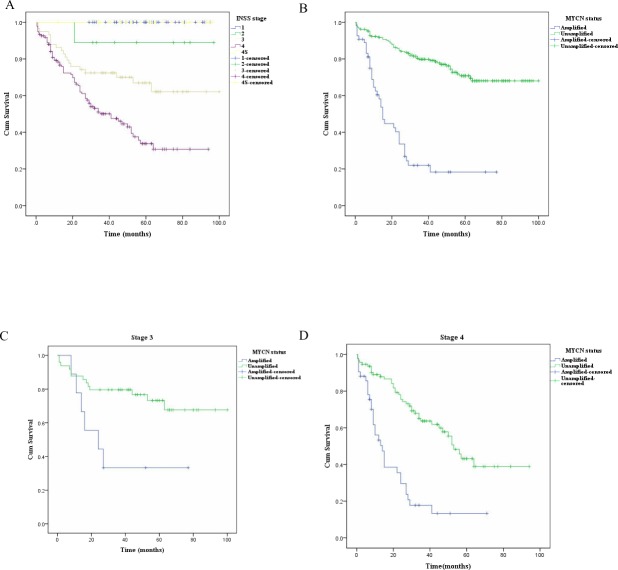
The Kaplan–Meier survival curves for NB patients (*n* = 262) with different stages and different MYCN status, stage 3 patients (*n* = 58) with different MYCN status, and stage 4 patients (*n* = 135) with different MYCN status. (A–B) The differences in Kaplan–Meier survival curves for NB patients (*n* = 262) with different stages (A) and different MYCN status (B) showed statistical significance (both *P* < .001 by log-rank test). (C–D) The comparison of Kaplan-Meier survival curves for different MYCN status groups at stage 3 (C, *n* = 58) and 4 (D, *n* = 135) had statistical significance (*P* = .012 and *P* < .001 by log-rank test, respectively).

Thirteen patients were excluded from the analysis of follow-up because they had rejected further treatment after definite diagnosis by biopsy or GTR, so the analysis of follow-up was a summary of 262 patients. The follow-up periods ranged from half a month to 100 months (average 40.6 months and median 40.0 months). The three-year and five-year OS rates are listed in Table 2 and they were lower for stage 3 and 4 patients. The median OS estimate was 15.0 (±1.6) months (95% CI [11.8–18.2]) for amplified group. The differences among Kaplan–Meier survival curves for different stages or different MYCN status (unamplified vs. amplified) were statistically significant (both *P*  <  .001 by log-rank test) (Figs. 2A and 2B ). The median OS estimate for MYCN amplified group was 24.0 (±11.9) months (95% CI [0.6–47.4]) for stage 3 patients. The median OS estimates for amplified and unamplified groups at stage 4 were 14.0 (±2.3) months (95% CI [9.4–18.6]) and 53.0 (±3.7) months (95% CI [45.7–60.3]), respectively. The differences among Kaplan–Meier survival curves for different MYCN status groups at stages 3 or 4 had statistical significance (*P* = .012 and *P*<.001 by log-rank test, respectively) (Figs. 2C and 2D). As for the three-year and five-year OS rate estimates the stage 3 and 4 patients still had the worst performance. The three-year and five-year survival rate estimates were 79.8% and 70.8% for the MYCN unamplified group, and 22% and 18.3% for the amplified group, respectively.

**Table 2:** The three-year and five-year OS rates of 262 NBs in different INSS stages.

INSS Stage	No	3-year OS rate	5-year OS rate
1	44	100%	100%
2	9	88.9%	88.9%
3	58	72.4%	66.9%
4	135	50.1%	33.8%
4S	16	100%	100%

**Notes.**

Abbreviations OS overall survival NB neuroblastoma INSS International Neuroblastoma Staging System

**Figure 3:**
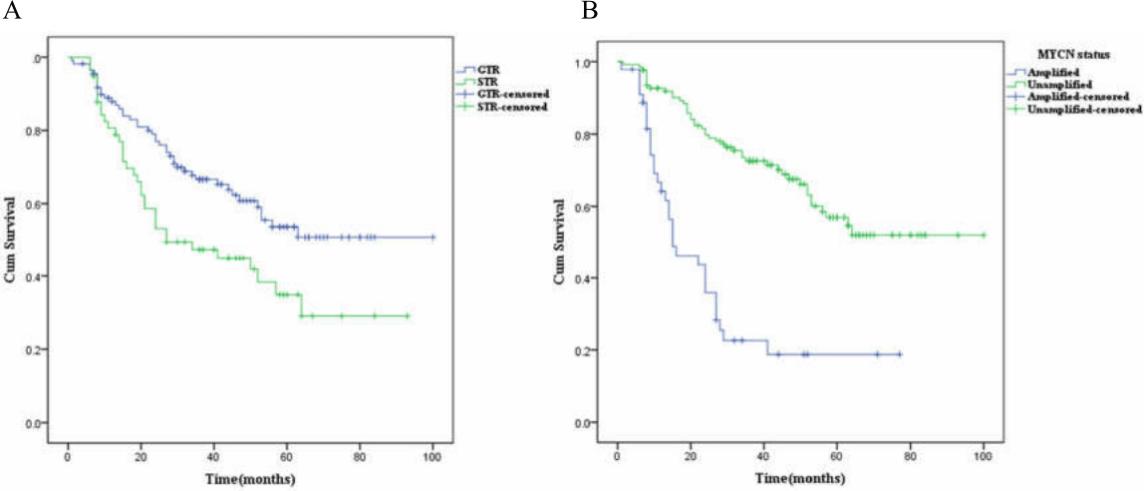
The Kaplan-Meier survival curves in the 169 stage 3 and 4 NB patients with different surgical methods (STR vs. GTR) and different MYCN status. (A–B) The differences in Kaplan–Meier survival curves for (A) different surgery groups and (B) different MYCN status showed statistical significance (*P* = .009 and *P* < .001 by log-rank test, respectively) in the 169 stage 3 and 4 patients.

The median OS estimates for STR group and MYCN amplified group for 169 stage 3 and 4 patients were 27.0 (±11.6) months (95% CI [4.2–49.8]) and 15.0 (±4.9) months (95% CI [5.4–24.6]), respectively. The differences between Kaplan–Meier survival curves for different surgery groups or different MYCN status groups had statistical significance (*P* = .009 and *P* < .001 by log-rank test, respectively) in those patients (Figs. 3A and 3B). The univariate and multivariate analyses by Cox regression analyses were depicted in Table 3. The former analysis revealed statistically significant variables included INSS stage, risk group, grade of differentiation, FH/UH, MYCN status, and sugical methods. In the multivariate Cox model different surgical methods (STR vs. GTR) and different MYCN status (unamplified vs. amplified) still showed statistically significant differences (*P* = .047 and *P* = .001, respectively) and they were the independent variables influencing the OSs. STR had a high relative risk compared with GTR (HR = 1.590,95% CI [1.006–2.513]).

**Table 3:** Univariate and multivariate analyses of OS rates in the 169 stage 3 and 4 patients by Cox regression model.

Variable		Univariate analysis			Multivariate analysis		
		*P*	HR	95% CI	*P*	HR	95% CI
Age	12 months		1			1	
	12–60 months	.022	2.729	1.159–6.426	.544	.674	.188–2.410
	≥60 months	.039	2.594	1.049–6.415	.599	.691	.174–2.743
Sex (Female vs. male)		.709	.914	.570–1.466	.968	1.010	.616–1.657
Site	Adrenal gland		1				
	Mediastinum	.130	.524	.227–1.210			
	Others	.868	.918	.334–2.524			
INSS stage (4 vs. 3)		.002	2.451	1.408-4.266	.262	1.398	.778-2.512
Risk group(High-risk vs. intermediate-risk)		.000	10.005	3.140–31.873	.007	7.829	1.752–34.983
Grade of differentiation	Undifferentiated subtye		1			1	
	Poorly differentiated subtype	.005	.519	.330–.817	.794	.931	.546–1.587
	Differentiating subtype	.035	.118	.016–.863	.807	.735	.062–8.695
FH/UH (FH vs UH)		.003	.313	.143–.682	.874	1.110	.308–3.996
MYCN status (Unamplificaction vs. amplification)		.000	.247	.154–.394	.001	.357	.196-.651
ASCT (No vs. yes)		.193	1.557	.799–3.032			
Radiation (No vs. yes)		.105	.678	.423–1.085			
Surgical methods(STR vs. GTR)		.011	1.794	1.143–2.817	.047	1.590	1.006–2.513

**Notes.**

Abbreviations HR hazard ratio CI confidence interval FH favorable histology UH unfavorable histology ASCT autologous stem cell transplantation STR subtotal resection GTR gross total resection

## Discussion

Most clinical studies into NBs have been conducted in Western countries or Japan, and the criterion on diagnosis, risk stratification, and treatment protocols was established by COG and INRG based on these studies. There is no specialized and collaborative research institute majoring in NB in China, and studies on a large series of Chinese NBs were uncommon. The three leading primary tumor sites were the adrenal gland (47%–50%), other abdominal sites (27%–29%), and thoracic sites (15%–16%) verified by two large series studies (Vo et al., 2014; Berthold et al., 2017). The main tumor original sites in our study were the adrenal gland (80.4%), thoracic sites (14.5%), and other abdominal sites (3.3%). The proportion of NBs at stage 4 in our center was 52% slightly higher than about 40% reported by the INRG database (Cohn et al., 2009). Most distant metastatic sites were bone and bone marrow consistent with other studies (Berthold et al., 2017; Morgenstern et al., 2016). This series supported most of the clinicopathological features published by previous studies, and we speculate that subtle differences may be partially attributable to geographical and racial factors.

MYCN status includes amplification, gain, and wild-type. MYCN amplification is a powerful prognostic factor for NBs regardless of other factors, such as age, staging, INPC, and genetic aberrations of 1p and 11q, and it has appeared in approximately 20% of NBs and about 38% of all stage 4 patients (Berthold et al., 2017; Maris, 2010). Two recent large series studies from COG and Germany showed the incidence of MYCN amplification in NBs was about 18% (Berthold et al., 2017; Campbell et al., 2017). The incidence of MYCN amplification in our series was about 21.5%, very close to that in the preceding studies. Its strong relationship with unfavorable histology, high-risk group, advanced stage, and poor OS rates in our study confirmed its significance in risk stratification and outcome prediction. However, Wang et al. (2013) reported that the proportion of MYCN amplification was 13.5% in another regional center in northern China, which suggested it might have regional diversity in Chinese NB patients. The two primary renal NBs in our study all had MYCN amplification. MYCN amplification in stage 1, 2, and 4S NBs is rare. Only three cases were observed in our series, and they often had a greater likelihood of relapse compared with the counterparts without MYCN amplification. Bagatell et al. (2009) reported 87 (3%) of the total 2660 NBs at stages 1 and 2 had MYCN amplified tumors, and they had less favorable OS than those with non-amplified tumors. No consensus has been reached regarding the significance of MYCN gain. Campbell et al. (2017) reported 133 (2.8%) of 4672 NBs had MYCN gain, and MYCN gain was associated with an inferior prognosis. However, different results were reported by Wang et al. (2013), Souzaki et al. (2011) and Spitz et al. (2004). The amount of MYCN gain in our series was so small that we could not derive any useful information.

Surgery is the mainstay for stage 1 and stage 2 NBs, and GTR has shown satisfactory outcome in these types of patients. This has been demonstrated in many studies, including this one (Pinto et al., 2015). For stage 4S patients, primary tumor resection is not obligatory because other treatment strategies such as wait-and-see and low-dose chemotherapy alone after biopsy can also be effective. There is no clear gap in the OS rates at all stages between our study and previous reports. However, the five-year OS rate in patients with MYCN amplification (most at stages 3 and 4) was only 18.3% in our study, far lower than that in previous studies (33%–34% for Japan and Germany). This is the core subgroup which should be dealt with to improve the OS in NBs (Berthold et al., 2017). The quick introduction of novel drugs and treatment methods may help to solve this problem.

Regarding surgery for stage 3 and 4 patients, there are no guidelines covering the choice of different surgical methods (STR vs. GTR), and the role of surgery on the improvement of OS in these patients is still debatable. GTR may be a challenge in some stage 3 and 4 patients because of the poor general situation, vascular encasement by the tumor, and invasion of adjacent organs. Aggressive operations are usually accompanied with potential high risk and may be life-threatening. There are many difficulties in estimating the role of surgery in stage 3 and 4 NBs, such as lack of available prospective studies, non-standardized assessment on the extent of surgical resection, differences in surgeons’ technical skills, and differences among hospital situations. The single-center study usually has a relatively stable surgical team and steady description of surgical approach but a small case number. However, multicenter studies may have the opposite advantages and disadvantages. Several early studies have shown that GTR could improve the prognosis, but they were often single-center studies and had a small case number, and miscellaneous factors were usually undiscussed (McGregor et al., 2005; La Quaglia et al., 2004; Escobar et al., 2006). However, recent studies have drawn the opposite conclusion. Simon et al. (2013) made a study of 278 stage 4 NBs aged 18 months or older from a German clinical trial and found primary tumor resection had no impact on local control rate and outcome. The radicality of surgery was not found to be significantly associated with better OS in stage 3 and 4 patients in Adeline’s study (Salim et al., 2011). What should be noticed is that new and effective chemotherapy/radiation regimens and the application of novel treatment protocols may conceal the real effects of GTR in stage 3 and 4 patients. The median OS estimate of the GTR group was longer than that of the STR group, the statistical significance of the Kaplan–Meier survival curves was determined by the log-rank test in our study, and multivariate survival analyses showed different surgical methods (STR vs. GTR) still had statistically significant difference under the Cox regression model with the adjustment of other variables, such as INSS stage, risk group, and MYCN status. Our study provides evidence supporting the positive conclusion and skilled surgeons should make great efforts to achieve GTR in stage 3 and 4 NB patients. More standardized studies from both single and multiple centers are needed to verify the effect of GTR in stage 3 and 4 patients and provide surgical guidelines for the treatment strategies.

Our study has some limitations. First, this is a single-center retrospective study with a relatively small number of NB patients, so a large-scale multicenter study is required to verify our findings. Second, there may be selective bias in the percentage of MYCN amplification in our NB patients. Last, the number of patients lost to follow-up may have some effects on the mean survival estimates and the comparison of survival rates for the GTR group and STR group in stage 3 and 4 patients.

## Conclusions

In summary, we report a large series of NBs in Eastern China and make a comprehensive study about the clinicopathological features, MYCN status, surgical management, and follow-up. We find that MYCN amplification is an independently adverse prognostic factor in Chinese NB patients at stages 3 and 4. GTR is closely associated with improved OS compared with STR in these types of patients, which may guide the optimal choice of surgical methods and a balance of risk and resection extent in these advanced-stage patients. The OS of stage 4 NBs with MYCN amplification is extremely poor, which may merit more detailed risk stratification and more effective treatment strategies.

##  Supplemental Information

10.7717/peerj.5665/supp-1 Supplemental Information 1 The raw data of Kaplan–Meier survival curves for 262 NB patients with different stages Outcome: 1, dead of disease, 0, alive or lost follow-up.

10.7717/peerj.5665/supp-2 Supplemental Information 2 The raw data of Kaplan–Meier survival curves for NB patients (*n* = 262) with different MYCN status MYCN status: N, unamplified, A, amplified;
Outcome:1, dead of disease, 0, alive or lost follow-up.

10.7717/peerj.5665/supp-3 Supplemental Information 3 The Kaplan–Meier survival curves for stage 3 NB patients (*n* = 58) with different MYCN status Outcome:1, dead of disease, 0, alive or lost follow-up;
MYCN status;N, unamplified, A, amplified.

10.7717/peerj.5665/supp-4 Supplemental Information 4 The Kaplan–Meier survival curves for stage 4 patients (*n* = 135) with different MYCN status Outcome:1, dead of disease, 0, alive or lost follow-up; MYCN status:U, unamplified, A, amplified.

10.7717/peerj.5665/supp-5 Supplemental Information 5 The raw data of Kaplan-Meier survival curves in the 169 stage 3 and 4 NB patients with different surgical methods (STR vs. GTR) and different MYCN status, and univariate and multivariate analyses of OS rates in these patients Age: 0, <12 months, 1, 12–60 months, 2, ≥60 months;
Sex:0, male, 1, female;
Site:0, adrenal gland, 1, mediastinum, 2, others;
Stage:0, 3, 1, 4
; Risk:0, intermediate-risk, 1, high-risk;
Outcome: 1, dead of disease, 0, alive or lost follow-up;
Autologous stem cell transplantation:0, no, 1, yes;
Radiation: 0, no, 1, yes;
Degree of differentiation:0, undifferentiated subtype, 1, poorly differentiated subtype, 2, differentiating subtype;
UH or FH: 0, UH,1, FH;
MYCN status: 0, amplified, 1, unamplified;
Surgical methods: 0, gross total resection, 1, subtotal resection.

## Additional Information and Declarations

### Data Availability

The following information was supplied regarding data availability: The raw data are provided in the Supplemental Files.
